# Epigenetic Regulation of the Wnt/β-Catenin Signaling Pathway in Cancer

**DOI:** 10.3389/fgene.2021.681053

**Published:** 2021-09-06

**Authors:** Ankita Sharma, Rafeeq Mir, Sanjeev Galande

**Affiliations:** ^1^Centre of Excellence in Epigenetics, Department of Biology, Indian Institute of Science Education and Research, Pune, India; ^2^Centre for Interdisciplinary Research and Innovations, University of Kashmir, Srinagar, India; ^3^Department of Life Sciences, School of Natural Sciences, Shiv Nadar University, Greater Noida, India

**Keywords:** Wnt signaling, β-catenin, YAP, epigenetics, cancer, therapeutics

## Abstract

Studies over the past four decades have elucidated the role of Wnt/β-catenin mediated regulation in cell proliferation, differentiation and migration. These processes are fundamental to embryonic development, regeneration potential of tissues, as well as cancer initiation and progression. In this review, we focus on the epigenetic players which influence the Wnt/β-catenin pathway via modulation of its components and coordinated regulation of the Wnt target genes. The role played by crosstalk with other signaling pathways mediating tumorigenesis is also elaborated. The Hippo/YAP pathway is particularly emphasized due to its extensive crosstalk via the Wnt destruction complex. Further, we highlight the recent advances in developing potential therapeutic interventions targeting the epigenetic machinery based on the characterization of these regulatory networks for effective treatment of various cancers and also for regenerative therapies.

## Introduction

The Wnt signaling pathway remains one of the most extensively studied signaling pathways to date and yet there is a long way to fully understand its functionality. The Wnt pathway was discovered in 1982 with the identification of the *int1* (*wnt1*) gene responsible for tumor growth (Nusse and Varmus, 1982). The hallmark Wnt pathway gene *wnt1* (*int1* at the time of identification) was discovered as the first proto-oncogene in mice mammary tumors using the pro-viral tagging screening method in 1982 (Nusse and Varmus, 1982). The Wnt pathway is associated with the widest array of biological processes, including cell proliferation, differentiation, organogenesis, regeneration and diseases such as neurodevelopmental diseases and cancer (Nusse and Clevers, 2017). Although the mammalian *wnt1* gene was discovered as a part of screening for proto-oncogenes, its *Drosophila* homolog *wingless* was already known to play a role in segmentation during development (Nüsslein-Volhard and Wieschaus, 1980). Other genes in this pathway (including, *armadillo, arrow, disheveled, zeste-white 3, porcupine*) were also identified due to segmentation polarity defects during *Drosophila* development (Peifer et al., 1991; Siegfried et al., 1992; Noordermeer et al., 1994; Kadowaki et al., 1996; Bafico et al., 2001). Overexpression studies of Wnt1 and GSK3β in *Xenopus* showed axis duplication (McCrea et al., 1993; Dominguez et al., 1995). Studies in planaria and *Hydra* have established the role of the Wnt pathway in regeneration as well (Hobmayer et al., 2000; Gurley et al., 2008; Iglesias et al., 2008; Petersen and Reddien, 2008; Reddy et al., 2020). Due to the important roles of the Wnt pathway in the processes of development and regeneration, the interest in investigating its role in human cancers climbed steadily. *APC* was found to be the most frequently mutated gene in inherited forms of human cancer, causing multiple polyps in intestines (Familial Adenomatous Polyposis or FAP) (Su et al., 1992). Sporadic cases of cancers, on the other hand, didn’t show alterations in the *APC* gene. Rather, other components of the Wnt pathway, such as *CTNNB1* and *Axin* showed mutations. Apart from genetic alterations in the Wnt pathway components, there are several epigenetic changes associated with tumor initiation and progression in Wnt driven cancers. Additionally, there are epigenetic changes which are a result of Wnt activation. There are three modes of mechanism for the epigenetic machinery to work. It can change the status of DNA methylation, histone modification profiles and can also work through a plethora of non-coding RNAs (Dai et al., 2020). Even though these do not change the DNA sequence but these are heritable changes and thus can contribute extensively toward tumorigenesis. Very recently, drugs targeting epigenetic machinery have been adopted into the chemotherapeutic regime for cancers reflecting the importance of epigenetic modulation in cancers (Topper et al., 2020).

This review is specifically aimed at providing an overview of all well characterized epigenetic mechanisms which modulate the expression and function of the canonical Wnt pathway components and therefore affect Wnt target genes in various cancer types. In addition to this, the review will also cover advances made in the therapeutic interventions targeting the Wnt signaling pathway, especially through the epigenetic players.

## Wnt Signaling Network – The Recent Most Snapshot

Wnt signaling is broadly classified into canonical and non-canonical Wnt signaling based on β-catenin -dependent and independent responses, respectively (Semenov et al., 2007). Canonical Wnt signaling is an intricate pathway involving 19 Wnt ligands, 10 Frizzled (FZD) receptors and 3 Disheveled (DVL) proteins. The selective combination of Wnt-FZD generates a wealth of information, modulating the signaling outcome crucial for normal development, multitude of cellular processes and development of various diseases (Semenov et al., 2007; Dijksterhuis et al., 2015; Voloshanenko et al., 2017). In unstimulated cells, the transcriptional co-activator β-catenin is engaged by a large cytoplasmic destruction complex composed of Adenomatous polyposis coli (APC), Axis inhibition protein (AXIN), Glycogen synthase kinase 3 (GSK3) and Casein kinase 1 (CK1), inducing sequential phosphorylation of β-catenin at Serine 33, Serine 37, Serine 45, and Threonine 41 by CK1 and GSK3β. The phosphorylated β-catenin is then ubiquitinated by βTrCP, an E3 ubiquitin ligase, followed by its degradation, preventing its nuclear transport (MacDonald et al., 2009). The stability of the destruction complex is very critical for continuous degradation of β-catenin. In canonical signaling, Wnt ligands bind to Frizzled and low-density lipoprotein receptor related proteins (LRP5/6) to stimulate Wnt signaling by recruiting the DVL proteins to the plasma membrane. Binding of DVLs to the membrane and sequential phosphorylation on the cytoplasmic domain of LRP5/6 promotes its interaction with AXIN, thereby destabilizing the destruction complex and inducing dis-engagement of β-catenin from the destruction complex. Moreover, the inactivation of ubiquitination and proteasomal degradation of β-catenin in the intact destruction complex have been shown to saturate the destruction complex, preventing further engagement and thereby degradation of β-catenin (Li et al., 2012). Disengaged β-catenin evades phosphorylation due to the multivesicular sequestration of GSK3β which prevents recognition by βTrCP E3 ubiquitin ligase and hence the degradation by the proteasome pathway (Taelman et al., 2010). Dephosphorylated β-catenin stabilizes and accumulates in the cytoplasm with subsequent translocation into the nucleus (MacDonald et al., 2009). Nuclear β-catenin interacts with the transcription factors of the TCF/LEF (T cell factor/lymphoid enhancer-binding factor) family by replacing corepressor, Groucho, to induce expression of Wnt/β-catenin target genes (Daniels and Weis, 2005).

**FIGURE 1:**
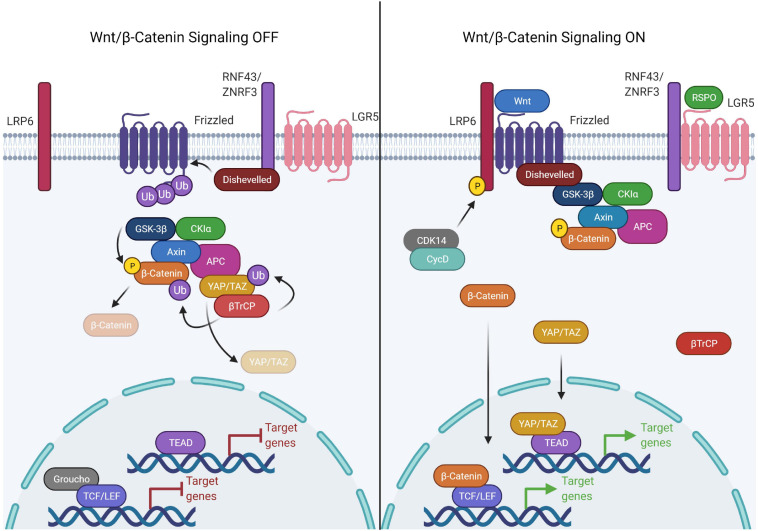
Components of the canonical Wnt signaling pathway. The canonical Wnt pathway is primarily dependent on the transcriptional activity of β-catenin. In the Wnt OFF state, the Wnt destruction complex is actively engaged in the phosphorylation and subsequent ubiquitination mediated degradation of β-catenin. This is achieved by sequestering β-catenin to the Wnt destruction complex, consisting of APC, AXIN (scaffold proteins), GSK3β, and CK1α (kinases). Upon phosphorylation by the kinases, β-catenin is ubiquitinated by βTrCP which is recruited to the destruction complex by YAP/TAZ. The DNA binding partner of β-catenin, TCF/LEF, is bound to the DNA in a repressive complex with Groucho/TLE. Wnt signaling is activated upon binding of Wnt ligand causing complex formation between LRP6 and frizzled which allows Disheveled to bind to LRP. This activated form of Disheveled is able to bind and recruit the Wnt destruction complex thereby disrupting the binding of βTrCP. The receptor bound Wnt destruction complex is ubiquitination incompetent, hence β-catenin can’t be targeted for proteasomal degradation, therefore leading to saturation of the destruction complex with phospho-β-catenin. The new pool of β-catenin is free to translocate into the nucleus and activate the transcription of Wnt responsive genes by binding the TCF/LEF family of transcription factors. In the same process, YAP/TAZ is unable to recruit β-TrCP to the destruction complex and escape degradation. YAP/TAZ translocates into the nucleus as well and binds to the TEAD family of transcription factors.

Multiple factors have been shown to regulate the precise stimulation and inhibition of Wnt signaling. For instance, the R-spondin/LGR/RNF43 module is the key signaling paradigm that potentiates Wnt signaling. R-spondin family proteins regulate Wnt signaling via a common mechanism involving interaction with the Leucine-rich repeat-containing G-protein coupled (LGR) family of receptors (Kim et al., 2008). In the absence of R-spondins, two homologous ubiquitin E3 ligases, ZNRF3/RNF43 bind to the FZD receptors and target it for degradation. However, in the presence of R-spondins, ZNRF3/RNF43 interact with LGR4-6, leading to the inhibition of ZNRF3/RNF43 and dickkopf glycoproteins family member, DKK1 activity allowing subsequent accumulation of FZD receptors, thereby potentiating Wnt signaling (Kim et al., 2008; Wang et al., 2013; Xie et al., 2013; Figure 1). A recent study suggests that LGR5 potentiates Wnt signaling without sequestering ZNRF3/RNF43 by directly enhancing the Wnt signalosome mediated LRP6 phosphorylation (Park et al., 2020). This finding establishes that LGR4 and LGR5 have non-equivalent functions adopting different routes to stringently potentiate Wnt signaling.

**FIGURE 2:**
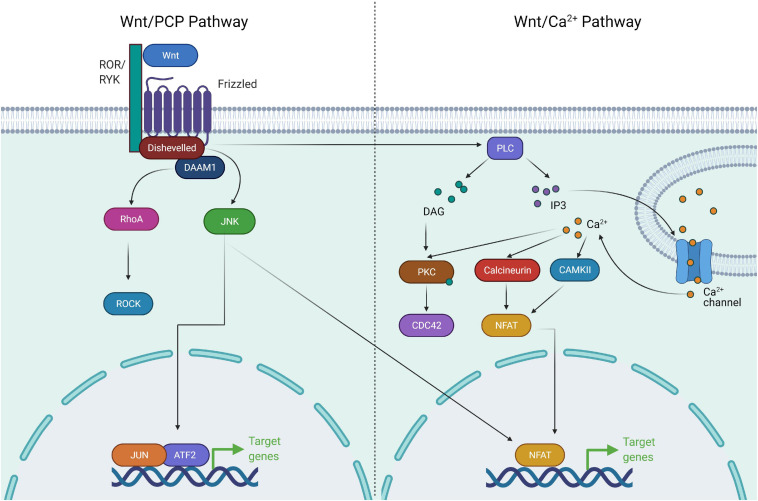
Components of non-canonical Wnt signaling pathway. The non-canonical Wnt pathway is independent of β-catenin. Wnt activation leads to complex formation between ROR and Frizzled which allows activation of Disheveled. Disheveled is then able to activate DAAM1 and RHOA GTPase. RHOA in turn activates ROCK. Activation of ROCK and JNK leads to cytoskeletal remodeling. Wnt/Ca^+^ signaling involves activation of Disheveled upon Wnt activation, followed by activation of PLC. PLC activates the downstream effectors PKC, Calcineurin, and CAMKII via release of Ca^+^. These factors drive NFAT mediated transcriptional program and cytoskeletal changes.

Non-canonical Wnt signaling, consisting of Planar cell polarity (PCP) and Ca^+^ signaling pathways, has been shown to converge with multiple pathways and regulate a diverse number of cellular processes required for development and organogenesis. The combination of receptor/co-receptor at the plasma membrane binding to Wnts acts as a regulatory switch to activate either β-catenin-dependent or β-catenin-independent pathways. In the PCP pathway, Wnt ligands, Wnt5a and Wnt11 bind to receptors FZD3 or FZD6 along with tyrosine kinase co-receptors ROR1, ROR2 or RYK to orient toward Wnt/PCP signaling (Oishi et al., 2003; Martinez et al., 2015). Subsequently, this leads to recruitment of DVLs, leading to the activation of small GTPases of the Rho family [Cdc42, Rac1, RhoA and DVL-associated activator of morphogenesis1 (DAAM1)] (Habas et al., 2001; Liu et al., 2008). This cascade leads to the activation of downstream c-Jun kinase and Rho associated kinase required for actin cytoskeletal remodeling and cell contractility (Sebbagh and Borg, 2014; Martinez et al., 2015). The Wnt/PCP pathway is also activated independent of DVLs and the cascade of signaling is transduced to activate Nemo like kinase (NLK) which is required for cell fate determination and cell movement (Thorpe and Moon, 2004). In the Wnt/Ca^+^ pathway, Wnt ligands bind to FZD with concomitant recruitment of DVLs leading to the production of intracellular signaling molecules, inositol 1,4,5-triphosphate (IP3), 1,2 diacylglycerol (DAG), and Ca^2+^ from the membrane-bound phospholipid phosphatidylinositol 4,5-bisphosphate via the action of membrane-bound enzyme phospholipase C (De, 2011). The network of this signaling paradigm leads to the activation of downstream signaling proteins such as protein kinase C, calcineurin and Ca^2+^/calmodulin-dependent protein kinase II (CaMKII), thereby regulating cell adhesion and cell migration (Malbon et al., 2000; Figure 2).

## Wnt Signaling in Cancers

Tumorigenesis involves progressive accumulation of genetic, epigenetic and molecular changes altering the cellular phenotype. Wnt signaling is a complex network of proteins and regulates molecular processes in a regulatory manner. Any imbalance in its regulation induces untoward cellular changes responsible for the development of various diseases and tumorigenesis. Dysregulation of Wnt signaling has been described in multiple cancers and plays a dramatic role in the progression of cancers (Bugter et al., 2021). Colorectal cancer is most commonly caused due to dysregulation of the Wnt pathway. It is sporadic although 30% of them are genetic in nature (Byrne and Tsikitis, 2018). Despite the role of the genetic mutations linked to colorectal cancer being well studied, the precise mechanism of the disease is not clearly understood. Mutations in the critical regulatory switches of the Wnt signaling pathway have been implicated in multiple cancers and loss of APC is a hallmark of colorectal cancer initiation and progression (Zhang and Shay, 2017). In most colorectal cancers, both the alleles of *APC* gene are mutated, especially in the region required for interaction with the armadillo (arm) repeats of β-catenin (Rubinfeld et al., 1997). Such mutations prevent the degradation of β-catenin and induce cellular changes critical for colorectal cancer initiation. Mutations in the *APC* gene are responsible for approximately 1% of all colorectal cancer cases (Bienz and Clevers, 2000; Half et al., 2009). The main feature of Wnt signaling driven cancers is the constitutive nuclear localization of β-catenin. Numerous studies have identified mutations in the serine and threonine residues of β-catenin causing abrogation of phosphorylation by GSK3β and CK1 kinases, ultimately leading to the hyperactivation of Wnt signaling (Sparks et al., 1998). Mutation in the serine/threonine regulatory domain of β-catenin has been found in 48% of colorectal cancers lacking *APC* mutations (Sparks et al., 1998). High throughput cancer genomics studies have cataloged mutations in the genes encoding multiple components of the Wnt signaling pathway, including the ligands, receptors and intracellular components. Besides *APC* and *CTNNB1*, mutations have been found in the regions of *AXIN1* gene coding for domains which are critical for its interaction with APC, GSK3β and β-catenin, resulting in destabilization of the destruction complex with concomitant increase in cytoplasmic as well as nuclear levels of β-catenin (Jin et al., 2003; Salahshor and Woodgett, 2005). Similarly, a mutation in *AXIN2* identified in colorectal cancers promotes GSK3β inhibition and stabilization of Snail, a positive regulator of Wnt signaling (Mazzoni et al., 2015). The increased expression of LRPs have also been linked with hyperactivation of Wnt signaling and development of colorectal cancers (Rismani et al., 2017; Boulagnon-Rombi et al., 2018; Yao et al., 2020). The R-spondin/LGR5/RNF43 module has also been implicated in Wnt signaling driven cancers. A deleterious mutation in *RNF43* has been reported in 19% of colorectal cancers lacking *APC* mutations (Giannakis et al., 2014). Further, a high number of R-spondin mutations and fusion proteins causing hyperactivation of Wnt signaling has been described in 10% of colon cancers lacking the *APC* mutations (Seshagiri et al., 2012; Giannakis et al., 2014).

Among gastric cancers, 30% exhibit nuclear β-catenin as a prominent acquired change (Clements et al., 2002). Around 18% of gastric cancers have mutations in *APC* and *RNF43* as the primary cause of the disease (Clements et al., 2002; Fang et al., 2002; Wang K. et al., 2014; Flanagan et al., 2019). In addition to the genetic mutations, epigenetic changes (such as promoter methylation) have also been identified in Wnt antagonists such as the secreted frizzled-related proteins (sFRPs) and Wnt negative-modulators, the dickkopf family of glycoproteins (DKK1–3), leading to activation of Wnt signaling (Nojima et al., 2007; Yu et al., 2009; Flanagan et al., 2017). These will be elaborated in the following sections.

In cancers, heterogenous levels of activation determine the adversity and fate of different cancers. For example, 37% of *APC*-mutant gastric cancers have mutation in *RNF43*, suggesting a compound activation of Wnt signaling in the same tumor. However, only 5.5% of colon cancers have mutations both in *APC* and *RNF43*, suggesting a differential mechanism of optimal activation of Wnt signaling in gastric and colon cancers for tumor growth and progression (Albuquerque et al., 2002). The role of Wnt signaling in hematopoietic stem cells and leukemia progression has also been described wherein β-catenin appears to be essential for leukemia initiating cells and their self-renewal (Albuquerque et al., 2002; Luis et al., 2012; Lento et al., 2013). Chronic lymphocytic leukemia shows higher expression of multiple Wnt proteins with enhanced β-catenin-dependent Wnt signaling (Lu et al., 2004). Further, epigenetic silencing of Wnt signaling inhibitors DKK1/2 and somatic mutations in *FZD5* is associated with the development of chronic lymphocytic leukemia in 14% of the cases (Moskalev et al., 2012; Wang L. et al., 2014). Table 1 summarizes the mutations in different Wnt components reported in various cancers. Recently, the synergistic interplay of the canonical and non-canonical Wnt signaling playing a major role in the development of cancers has also been established (Katoh, 2017; Flores-Hernández et al., 2020).

**TABLE 1:** Mutations in Wnt signaling components in cancers.

Gene	Alteration	Cancer type	Outcome	References
**APC**	Exon 15	CRC	Loss of interaction with β-catenin	Rubinfeld et al., 1997; Bienz and Clevers, 2000; Half et al., 2009
**APC**	Exon 15	GC	Loss of interaction with β-catenin	Fang et al., 2002; Flanagan et al., 2019
**β-catenin**	Codons 33, 37, 41, and 45 in exon 3	CRC	Loss of phosphorylation by GSK3β and Ck1α	Sparks et al., 1998
**AXIN1**	Exon 1 and 5	CRC	Loss of interaction with APC, GSK3β and β-catenin	Jin et al., 2003; Salahshor and Woodgett, 2005
**AXIN2**	Stop codon at codon 663 in exon 7	CRC	Inhibition of GSK3β and stabilization of Snail	Mazzoni et al., 2015
**RNF43**	Truncation mutations	CRC	Inactivation of RNF43 and increased cell surface abundance of FZD5	Giannakis et al., 2014
**RNF43**	Truncation mutations	GC	Inactivation of RNF43 and increased cell surface abundance of FZD5	Wang K. et al., 2014
**R-spondins**	Gene fusions	CRC	Gene fusion of R-spondins (RSPO2 and RSPO3) with PTPRK and/or EIF3E causing elevated expression of R-spondins	Seshagiri et al., 2012

## Convergence of Wnt Pathway With Other Signaling Networks in Cancer

Signaling pathways do not operate linearly nor exclusively. Rather, there are multiple points of crosstalk between all the pathways which ultimately fine tune target gene expression. The Wnt pathway is known to work in conjunction with other pathways to regulate the fundamental cellular processes critical for normal development and cancer.

### Wnt and Hippo Signaling Pathways: Partners in Action

Like the Wnt pathway, the Hippo signaling pathway was also identified in *Drosophila*. The pathway gets its name owing to the phenotype it induces in flies. Flies with mutations in the Hippo pathway components have abnormal growth due to loss of organ size control (Justice et al., 1995; Xu et al., 1995; Jia et al., 2003; Udan et al., 2003). Studying crosstalk between the Wnt and Hippo pathways has been of particular interest to researchers. It is due to the fact that the effector proteins of these pathways, β-catenin and YAP/TAZ, directly interact with each other and their stability is dependent on the Wnt destruction complex (Azzolin et al., 2012, 2014). The Hippo pathway does not consist of a defined set of receptors and has diverse upstream regulators (Pan, 2010; Totaro et al., 2018). Unlike other pathways, the activation of the Hippo pathway leads to inactivation of its effector molecules, YAP and TAZ. These regulators can either be components of other signaling pathways or, often, are part of the cell’s mechanosensory machinery (Zhao et al., 2007; Lei et al., 2008). The mechanosensory proteins are the ones that are involved in the maintenance of cell-cell adhesion, apico-basal polarity and cell junctions, for example, *E*-cadherin, KIBRA, NF2, α-catenin and the Crumbs complex (Genevet et al., 2010; Varelas et al., 2010b; Zhang et al., 2010; Silvis et al., 2011; Benham-Pyle et al., 2015). Proteins, such as NF2, act as a scaffold for the assembly of Hippo kinases (Lallemand et al., 2003). Activation of Hippo signaling leads to the activation of a cascade of kinases, namely, Macrophage-stimulating protein (MST1/2), Salvador (SAV1), Large tumor suppressor (LATS1/2), and Mps one binder kinase (MOB1). MST1/2 is the upstream kinase. It gets activated upon phosphorylation and forms an active complex with SAV1. This complex further activates LATS1/2 via phosphorylation. LATS1/2, along with MOB1 leads to the phosphorylation of YAP/TAZ (Chan et al., 2005; Callus et al., 2006; Zhao et al., 2007; Praskova et al., 2008; Varelas et al., 2010b). The phosphorylated form of YAP/TAZ is sequestered in the cytoplasm and subsequently degraded due to ubiquitination (Zhao et al., 2007, 2010; Lei et al., 2008). Absence of Hippo signaling allows nuclear accumulation of YAP/TAZ which then bind to the TEAD (transcriptional enhanced associate domain) family of DNA binding proteins, leading to transcription of its downstream targets (Vassilev et al., 2001; Mahoney et al., 2005). YAP/TAZ can have other DNA binding partners as well, such as p75, RUNXs and SMADs (Yagi et al., 1999; Strano et al., 2001; Varelas et al., 2008). Due to its involvement in regulating organ size control and cell adhesion properties, its role in multiple cancer types is not surprising. It has been reported to be involved in cases of breast cancer, lung cancer, liver cancer, prostate cancer, etc. (Chan et al., 2008; Zhou et al., 2011; Chen Q. et al., 2014; Ma et al., 2014; Chen H.-Y. et al., 2015; Seo et al., 2017). Similar to the Wnt signaling pathway, the Hippo pathway is involved in the regulation of intestinal regeneration and is therefore frequently dysregulated in colorectal cancers as well (Cai et al., 2010; Gregorieff et al., 2015). Interestingly, even though the Hippo pathway is frequently perturbed in various cancer types, mutations in its components are uncommon (Harvey et al., 2013).

**FIGURE 3:**
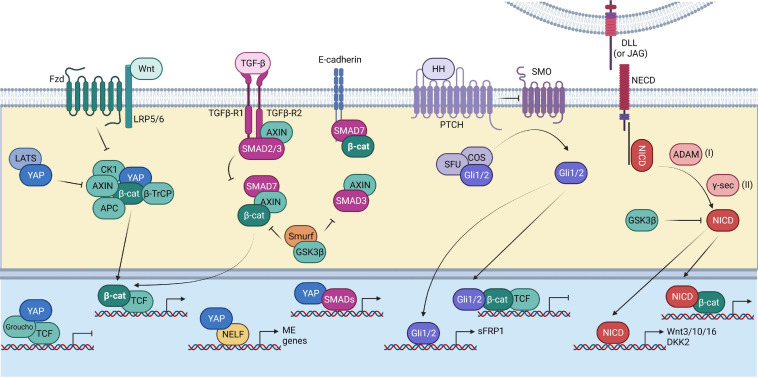
Interplay of the Wnt pathway with other pathways. Summary of the interaction of the Wnt pathway components (depicted in green) with other signaling pathways, including the Hippo pathway (depicted in blue), TGF-β pathway (depicted in magenta), Hedgehog pathway (depicted in purple), and Notch pathway (depicted in red). The Hippo pathway helps in the stabilization of β-catenin by inhibiting YAP’s interaction with the Wnt destruction complex, thereby preventing the recruitment of βTrCP. TGF-β signaling has synergistic effects with Wnt signaling. SMAD7 can help in preventing the GSK-3β mediated β-catenin degradation. The Hedgehog effector Gli1/2 can inhibit Wnt target gene expression by directly binding to the β-catenin/TCF complex. It can also drive the expression of the Wnt inhibitor sFRP1. The Notch signaling transcription factor, NICD, interacts with β-catenin. NICD can promote Wnt signaling by promoting the expression of the Wnt ligands. However, the Wnt inhibitor DKK2 is also a target of NICD. NICD itself is a substrate of GSK-β.

The first link between the Wnt and Hippo pathways was established in a study which showed TAZ as the negative regulator of the Wnt pathway. It was shown that TAZ inhibits interaction of DVL with CK1δ/ε thereby preventing its phosphorylation upon Wnt activation leading to inhibition of Wnt signaling (Varelas et al., 2010a). Recently, LATS1/2 have been shown to maintain the intestinal stem cell niche by sustaining Wnt signaling (Li Q. et al., 2020). The study shows that when the Hippo pathway is inactive, nuclear YAP/TAZ interacts with Groucho and prevents the Wnt mediated transcriptional program (Figure 3). Interestingly, LATS1/2 depletion was able to silence the expression of the Wnt target gene, *MYC*, even in the *APC* mutant intestinal cells, making it a good candidate for intestinal tumor therapy. Other evidence of positive regulation by the Hippo pathway showed that S127D mutant YAP (phosphomimetic form and hence constitutively cytoplasmic) inhibited the nuclear accumulation of β-catenin (Imajo et al., 2015). In contrast, few reports have suggested a negative role of the Hippo pathway in the regulation of Wnt signaling. A study showed that *Taz* knockout in mice kidney led to a minimal increase in nuclear β-catenin (Makita et al., 2008). An increased expression of β-catenin was observed in the case of cardiomyocytes from *Salvador* (*Sav1*) knockout mice (Heallen et al., 2011). Additionally, it was shown that YAP/TEAD and β-catenin/TCF complexes bind cooperatively to a few of their target genes, for e.g., *snail2* and *sox2* (Heallen et al., 2011). Most importantly, all the three effectors, β-catenin, YAP and TAZ, are involved in regulating βTrCP mediated ubiquitination and subsequent degradation of each other. YAP and TAZ themselves are part of the Wnt destruction complex (Azzolin et al., 2012). In Wnt off conditions, YAP/TAZ binds to the destruction complex and recruits βTrCP, thereby promoting degradation of β-catenin as well as YAP/TAZ (Azzolin et al., 2014). Similarly, β-catenin phosphorylated by GSK3β can bind to TAZ and initiate its ubiquitination (Azzolin et al., 2012). A recent report showed that the balance between β-catenin and YAP is crucial for the maintenance of the intestinal stem cell niche. YAP overexpression or LATS depletion resulted in the inhibition of Lgr5^+^ intestinal stem cell growth by re-programming these cells into a state of low Wnt and high Klf6, driving the cells toward a ‘wound-healing state’. The same study showed a similar effect in cases of organoids, patient derived xenografts as well as colorectal cancer cell lines (Cheung et al., 2020).

### TGF-β Signaling Pathway

The interplay between TGF-β and Wnt pathways is context-dependent and can result in different outcomes. For example, Wnt3a stimulation increases TGF-β expression as well as SMAD2 phosphorylation and promotes differentiation in the case of myofibroblasts (Carthy et al., 2011). Wnt and TGF-β activation have synergistic effects during EMT in alveolar epithelial cells (Zhou et al., 2012). TGF-β and Wnt activation promotes expression of mesendodermal (ME) related genes in human embryonic stem cells (hESCs). YAP recruits Negative elongation factor (NELF) and inhibits the expression of ME related genes (Estarás et al., 2015). Inhibitory SMAD7 promotes β-catenin binding to plasma membrane through *E*-cadherin and prevents its degradation in cancer epithelial cells and helps in regulation of cell–cell adhesion. Complex formation between SMAD7 and AXIN prevents interaction of β-catenin with GSK3β and E3 ligase Smurf2 via AXIN. This prevents degradation of β-catenin but increases its localization on the membrane instead of nucleus (Tang et al., 2008). On the other hand, an earlier study showed that SMAD7 can promote the nuclear translocation of β-catenin in prostate cancer cells (Edlund et al., 2005). AXIN proteins have been shown to play a contrasting role in the regulation of TGF-β signaling. Earlier studies showed that AXIN acts as a positive regulator of TGF-β signaling by promoting phosphorylation of SMAD3 upon TGF-β activation (Furuhashi et al., 2001; Dao et al., 2007). However, another report showed that AXIN is able to mediate binding of SMAD3 (not SMAD2) to GSK3β and promotes SMAD3 phosphorylation and degradation in the absence of TGF-β signaling (Figure 3). Depletion of AXIN increased the transcriptional activity of SMAD3 even in the absence of TGF-β activation (Guo et al., 2008). As mentioned earlier, SMADs are known DNA-binding partners of YAP/TAZ, apart from the TEAD proteins. YAP and TAZ interact with activated SMAD2/4 and SMAD3/4 complexes and increase their nuclear localization (Hiemer et al., 2014). TGF-β activation also leads to an increase in TAZ mRNA and not YAP, in a SMAD3-independent manner (Miranda et al., 2017). TAZ has been shown to enhance the metastatic properties of breast cancer cells via expression of BMP4 and subsequent activation of BMP signaling (Lai and Yang, 2013). However, when the Hippo pathway is activated and YAP/TAZ accumulates in the cytoplasm, the nuclear translocation of SMADs is blocked (Barrios-Rodiles, 2005).

### Hedgehog Signaling

The crosstalk between Hedgehog and Wnt signaling is stage-specific. The inhibitory feedback between the two pathways is responsible for driving developmental processes. Canonical Hedgehog signaling is an inhibitor of Wnt signaling especially in the context of intestinal homeostasis. Hedgehog signaling suppresses Wnt signaling by regulating the expression of sFRP1 via its effector proteins, Gli1 and Gli2 (He et al., 2006; Figure 3). Activation of Hedgehog signaling prevents the nuclear accumulation of β-catenin in liver cancers as well (He et al., 2006). However, the non-canonical Hedgehog signaling, which is Patched1 (PTCH1)-dependent and Smoothened (SMO) and Gli-independent, has been shown to positively regulate Wnt signaling in the intestinal tumors by promoting the undifferentiated state of cancer stem cells (Regan et al., 2017). Interestingly, one study showed that the advanced stages of colon cancer have a higher activation level of Hedgehog and not Wnt signaling. Activation of Hedgehog signaling also led to the expression of embryonic genes such as, POU5F1 and NANOG as well as inhibition of β-catenin/TCF transcriptional activity via Gli1. Activation of embryonic genes therefore might be responsible for increasing cancer stemness and metastatic properties in Hedgehog driven cases (Varnat et al., 2010). In contrast, Gli2 was shown to promote nuclear localization of β-catenin and together these factors drive the proliferation of osteosarcoma cells (Ma J. et al., 2019; Xu et al., 2019). Sonic hedgehog has been reported to positively regulate YAP in medulloblastoma. It not only drives the expression of YAP but also promotes its nuclear accumulation (Fernandez-L et al., 2012). However, in case of the intestinal mesenchyme, YAP/TAZ promote proliferation and self-renewal whereas Hedgehog signaling drives differentiation into smooth muscle (Cotton et al., 2017).

### FGF Signaling

The Wnt-FGF gradient is a paradigm which has been very well established in the context of development. It plays an extensive role during axis formation in development (Dyer et al., 2014; Oginuma et al., 2017). Due to similar reasons, the interplay between the two pathways is important in cancer as well. Wnt1 and Fgf3 expression is upregulated in cases of breast cancer. Breast cancer cell lines overexpressing both these ligands and therefore with high Wnt and Fgf activity, exhibited higher mitochondrial biogenesis which correlated with enhanced mammosphere forming capacity and stemness (Lamb et al., 2015). An extensive axis between Wnt and FGF regulates the stem cell niche in the lungs. In a study by Volckaert et al. (2017), it was shown that YAP expression promotes the basal stem cell niche in the lung epithelium. Activation of the Hippo pathway keeps the differentiated epithelium in a quiescent state. However, injury leads to the activation of YAP caused due to the degradation of Merlin, an upstream regulator of the Hippo pathway. Yap in turn causes induction of Wnt7b which in turn drives the expression of FGF10 which is required for the maintenance of basal stem cells (Volckaert et al., 2017).

### Notch Signaling

Notch activity is Wnt-dependent and it cooperates with Wnt in development as well as cancer. Cancer stem cells isolated from clear cell renal cell cancer specifically showed upregulation of the Wnt and Notch related genes including *TCF7L2*, *TCF3*, *LGR4*, *AXIN2*, *EP300RBPJ*, *NOTCH3*, *HES1*, and *JAG1* (Fendler et al., 2020). At a mechanistic level, it has been shown that the Notch intracellular domain (NICD) is a substrate for GSK3 kinases (Espinosa et al., 2003). The activity of GSK3-β is inturn dependent on the Wnt activation status. In Wnt inactive state, GSK3-β-dependent phosphorylation primes Notch for proteasomal degradation, thereby preventing its transcriptional activity (Figure 3; Espinosa et al., 2003). β-catenin can directly bind to NICD and enhance its transactivation. However, overexpression of LEF1 hinders this process by competing with NICD to interact with β-catenin (Jin et al., 2009). In another study, it was shown that Notch signaling is required for maintaining the stemness of mammary associated stem cells. Notch activation through stem cells in macrophages eventually led to expression of multiple Wnt ligands- Wnt3, 10, and 16 which ultimately promoted self-renewal in stem cells (Chakrabarti et al., 2018). Wnt signaling has been shown to prevent differentiation of hESCs into Medial Ganglionic Eminence (MGE)-like cells and promoted expansion of progenitors by driving the expression of the Notch ligand, JAG1 (Ma L. et al., 2019). However, it has also been shown that *DKK2* is a target of Notch signaling in the intestinal stem cells, indicating a negative feedback for Wnt by Notch signaling (Katoh and Katoh, 2007).

### Transcription Factors

Wnt signaling pathway partners with various transcriptional factors and chromatin remodelers, mainly via interaction with β-catenin, presumably assisting the expression of target genes for these transcription factors in a Wnt-dependent manner. A few instances have been reported in which these transcription factors can act as negative regulators of Wnt signaling as well. Some of these include SOX17, CBP, FOXO, MLL1, TBP, and HIF1a. A number of these factors are reviewed in Valenta et al. (2012) and Söderholm and Cantù (2020). In this review, we have focused on the two protein families that have been recently identified to work alongside the Wnt pathway.

The Homeodomain (HOX) group of proteins are involved in embryogenesis. In adults, these are required for maintenance and homeostasis. HOXA5 has been shown to coordinate with the Wnt pathway in driving stem cell differentiation. HOXA5 acts as a tumor suppressor and promotes intestinal cell differentiation by inhibiting the Wnt/β-catenin pathway. Wnt pathway, on the other hand, inhibits HOXA5 via MYC which is a direct target of the Wnt signaling (Ordóñez-Morán et al., 2015). Another study using cervical cancer cells showed that HOXA5 can inhibit Wnt pathway genes by transactivating TP53. TP53 regulates the expression of the miR-200 family of microRNAs which target multiple Wnt pathway components, as mentioned in the proceeding sections (Ma et al., 2020). In contrast, HOXA13 exerts an opposite effect. It binds to β-catenin and helps in the nuclear translocation and subsequent activation of β-catenin/TCF/LEF mediated transcription (Gu et al., 2020). HOXB4 is another HOX gene family member which appears to have both tumor suppressive and oncogenic potential depending on the cancer type. In a recent study, it was shown to inhibit cancer cell proliferation and further tumorigenesis by inhibiting β-catenin at the transcriptional level by binding to its promoter in cervical cancer cells. Interestingly, several other Wnt related genes were found to be downregulated in cells overexpressing HOXB4, including *MYC*, whereas genes such as *FZD8*, *DKK1*, and *AXIN2* were upregulated (Lei et al., 2021).

The Specificity Protein (SP) family of zinc finger proteins have been shown to regulate Wnt signaling as well. SP1, one of the earliest transcription factors to be identified, has been shown to regulate the stability of β-catenin by modulating its interaction with the Wnt destruction complex. Interestingly, SP1 depletion led to an increased phosphorylation and ubiquitination of β-catenin in colorectal cancer cell lines (Mir et al., 2018). SP5, on the other hand, acts as a feedback inhibitor of Wnt signaling. Its expression is induced upon Wnt activation, especially in cells with stemness, including embryonic carcinoma cells (Huggins et al., 2017). Further, Huggins et al. (2017) hypothesized that SP5 might compete with SP1 for binding to a limited set of loci upon Wnt activation in case of human pluripotent stem cells. These loci are mainly Wnt target genes which are downregulated by SP5 (Huggins et al., 2017). Interestingly, SP5 is also strongly expressed in Lgr5^+^ intestinal stem cells (Barker et al., 2010). Another study showed that SP5 and SP8 both assist the binding of β-catenin to LEF1 upon Wnt activation. This study also pointed out that SP5 and SP8 are responsible for regulating only a subset of Wnt target genes (Kennedy et al., 2016).

## Wnt Signaling and Epigenetic Regulation

### Modes of Epigenetic Regulation

Cells in multicellular organisms are homogenous at the genetic level but their phenotypic heterogeneity is a key hallmark at the structural and functional level. This heterogeneity is considered crucial in the regulation of gene expression and such heritable alterations that do not affect the DNA sequence are referred to as epigenetic changes. The epigenetic pathways respond to various signaling cues such as DNA methylation, histone variants, histone modifications, chromatin structure, nucleosome remodeling, and epigenetic interactions (Allis and Jenuwein, 2016). DNA methylation is one of the major epigenetic modifications regulating gene expression by altering DNA conformation, chromosome structure and by recruiting other epigenetic regulators (Denis et al., 2011; Hervouet et al., 2018; Laisné et al., 2018). DNA methylation is catalyzed by three methyltransferases DNMT1, DNMT3a and DNMT3b. DNMT3a and DNMT3b catalyzes *de novo* methylation while DNMT1 is required for maintenance and is critical for the inheritance of DNA methylation (Moore et al., 2013; Greenberg and Bourc’his, 2019). Around 1.5% of the human genome is methylated and the majority of methylation occurs at CpG sites on gene promoters. Aberrant hypermethylation at CpG sites is associated with diverse cancers (Robertson, 2001).

The selective incorporation of histone modifications at gene promoters and enhancers acts as a regulatory switch to determine the fate of gene expression. Trimethylation of histone H3 at lysine 4 (H3K4me3) is enriched at the active promoters whereas trimethylation of histone H3 lysine 27 (H3K27me3) and histone H3 lysine 9 (H3K9me3) are typically seen at inactive promoters. The enhancers are enriched with histone H3 lysine 4 monomethylated (H3K4me) and acetylated histone H3 lysine 27 (H3K27Ac) modifications. The histone methyltransferases (KMTs) and histone demethylases (KDMs) add and remove methyl groups to/from histones respectively. Histone acetyltransferases (HATs) and histone deacetylases (HDACs) add and remove acetyl groups to/from histones, respectively (Hon et al., 2009; Hawkins et al., 2011; Albini et al., 2019). The incorporation of these modifications involves dynamic regulation and any deviation from the regulation is associated with developmental defects and diseases (Hon et al., 2009; Hawkins et al., 2011).

MicroRNAs (miRs) are small non-coding RNAs typically consisting of 20–21 nucleotides and are essential for various developmental processes and diseases. They regulate gene expression post-transcriptionally either by inhibiting translation or inducing mRNA degradation (Filipowicz et al., 2008). Another group of non-coding RNAs called the long non-coding RNAs (lncRNAs) with less protein coding potential (also referred as transcription noise) are 200 nucleotide long and have been shown to be essential for regulating transcription, translation, splicing, chromatin modification and structure (Jarroux et al., 2017; Kopp and Mendell, 2018).

### Epigenetic Regulation of Wnt Signaling Components

Wnt signaling is a complex pathway modulated by various cellular and environmental cues to precisely regulate the myriad of cellular processes during development and disease. Wnt driven cancers have either hyperactivation of positive regulators or hypoactivation of negative regulators. In this section, we provide the details on the epigenetic modulations undergone by the components of the Wnt pathway. Table 2 summarizes the epigenetic regulation of Wnt components discussed in the following section.

**TABLE 2:** Epigenetic mechanisms regulating the Wnt components in different types of cancer.

Wnt component	Epigenetic alteration	Cancer type	Outcome	References
**Wnt2**	‘Loss of H3K27me3	CRC	Upregulation	Jung et al., 2015
	miR-548b downregulation	CRC	Upregulation	Xu et al., 2020
**Wnt5a**	Promoter hypermethylation	CRC	Downregulation	Ying et al., 2008
**Wnt9a**	Promoter hypermethylation	CRC	Downregulation	Galamb et al., 2016
**Wnt10b**	Promoter hypermethylation	CRC	Downregulation	Yoshikawa et al., 2007
**DKK1**	Promoter hypermethylation	CRC, CLL, BC	Downregulation	Nojima et al., 2007; Yu et al., 2009; Felipe de Sousa et al., 2011; Moskalev et al., 2012; Flanagan et al., 2017
	loss of H3Ac	Glioma, BC	Downregulation	Kim H.-Y. et al., 2015
	loss of H3K4me3	BC	Downregulation	Huang et al., 2013
	miR-375 upregulation	BC	Downregulation	Taube et al., 2013
	miR-376 upregulation	BC	Downregulation	Cui et al., 2016
**DKK3**	Promoter hypermethylation	CRC, BC	Downregulation	Xiang et al., 2013
**sFRP1**	Promoter hypermethylation	CRC	Downregulation	Suzuki et al., 2002; Fujikane et al., 2010; Li et al., 2018
	miR-139-5p and miR-9 upregulation	BC	Downregulation	Yang et al., 2015
**sFRP2**	Promoter hypermethylation	CRC, HCC, BC	Downregulation	Suzuki et al., 2002; Veeck et al., 2008; Yu et al., 2019; Xiang et al., 2021
**sFRP4**	Promoter hypermethylation	CRC	Downregulation	Suzuki et al., 2002
**sFRP5**	Promoter hypermethylation	CRC	Downregulation	Suzuki et al., 2002
**WIF1**	Promoter hypermethylation	CRC, BC, OC	Downregulation	Ai et al., 2006; Paluszczak et al., 2015
	miR-603 downregulation	Glioma	Downregulation	Hu et al., 2018
				Guo et al., 2014
**Fzd4**	miR-505 downregulation	CC	Upregulation	Ma et al., 2017
	loss of H3K27me3	GC	Upregulation	Li Z.-T. et al., 2020
**Fzd6**	miR-199a-5p downregulation	CRC	Upregulation	Kim B.-K. et al., 2015
	miR-130-30p downregulation	BC	Upregulation	Poodineh et al., 2020
**Fzd7**	miR-613 downregulation	PC	Upregulation	Song et al., 2017
**Fzd8**	miR-100 downregulation	BC	Upregulation	Jiang et al., 2016
**Fzd10**	Gain of H3K9Ac	BC	Upregulation	Gong et al., 2014
**LRP6**	miR-34 downregulation	CRC, BC	Upregulation	Kim et al., 2011
	miR-130-30p downregulation	BC	Upregulation	Poodineh et al., 2020
	miR-487b downregulation	CRC	Upregulation	Hata et al., 2017
	miR-181 upregulation	OC	Upregulation	Ruan et al., 2020
**LGR5**	Promoter hypermethylation	CRC	Downregulation	Su et al., 2015
	miR-363 downregulation	CRC	Upregulation	Tsuji et al., 2014
	miR-34 downregulation	CRC	Upregulation	Hahn et al., 2013
**APC**	Promoter hypermethylation	CRC	Downregulation	Su et al., 1992, 1993; Arnold et al., 2004
	miR-135 upregulation	CRC	Downregulation	Segditsas et al., 2008; Liang et al., 2017
	miR-142 upregulation	BC	Downregulation	Nagel et al., 2008; Isobe et al., 2014
**AXIN2**	Promoter hypermethylation	CRC	Downregulation	Koinuma et al., 2006
	miR-34 upregulation	CRC	Downregulation	Kim et al., 2013
	miR-103/107 upregulation	CRC	Downregulation	Kim et al., 2013; Chen H.-Y. et al., 2019
**GSK3-β**	miR-224 upregulation	CRC	Downregulation	Li T. et al., 2016
	miR-1229 upregulation	BC	Downregulation	Tan et al., 2016
**βTrCP**	miR-182 upregulation	CRC	Downregulation	Wang S. et al., 2016
	miR-135b upregulation	OS	Downregulation	Jin et al., 2017
**CK1α**	miR-155 upregulation	CRC, BC	Downregulation	Zhang et al., 2012
	miR 135b upregulation	OS	Downregulation	Jin et al., 2017
**YAP1**	miR-506 downregulation	BC	Upregulation	Hua et al., 2015
	miR-506 upregulation	CRC	Downregulation	Krawczyk et al., 2017
	miR-1224/CREB promoter	HCC	Downregulation	Yang et al., 2021
	hypermethylation	BC	Downregulation	Real et al., 2018
**TAZ1**	LINC00174 upregulation	CRC	Upregulation	Shen et al., 2018
	miR-125a downregulation	CRC	Upregulation	Yang M. et al., 2019
**LATS1/2**	Promoter hypermethylation	CRC, HNSCC, BC, RCC	Downregulation	Okami et al., 2005; Steinmann et al., 2009; Chen et al., 2013; Wierzbicki et al., 2013; Chen K.-H. et al., 2014
**MST1/2**	Promoter hypermethylation	HNSCC, Mesothelioma	Downregulation	Steinmann et al., 2009; Maille et al., 2019
**β-catenin**	miR-200a downregulation	CRC	Upregulation	Tian et al., 2014
	miR-141 downregulation	BC	Upregulation	Abedi et al., 2015
	miR-340 downregulation	BC	Upregulation	Abedi et al., 2015
	miR-520f-3p downregulation	GC	Upregulation	Chen et al., 2020
**TCFL1/2**	Promoter hypomethylation	CRC	Upregulation	Guo et al., 2015
**TCF7**	miR-29 downregulation	CRC	Upregulation	Subramanian et al., 2014
**LEF1 (transcript from P2)**	miR-34	BC	Upregulation	Kim et al., 2011
	gain of H3K9me3	BC	Downregulation	Yokoyama et al., 2010
**TCF7**	LOC728196 upregulation	Glioma	Upregulation	Wang O. et al., 2018

#### Ligands

Several tumor types are dependent on the hyperactivation of Wnt ligands because it provides an advantage to the tumor cells. Wnt secretion not only activates autocrine signaling but can also act on neighboring cells via paracrine signaling (Carbone et al., 2018; Eyre et al., 2019). Cancer cells seem to utilize multiple mechanisms to upregulate the expression of Wnt ligands. For example, Wnt2 expression can be upregulated in colorectal cancer via loss of repressive histone mark H3K27me3 by EZH2 (Jung et al., 2015). Wnt2 expression is also enhanced by downregulation of its regulatory miRNAs. In case of colorectal cancer, miR-548b overexpression can suppress cell proliferation by targeting Wnt2 (Xu et al., 2020). Several upstream regulators can also be targeted to achieve higher expression of the Wnt ligands. miR-600 targets SCD1 (stearoyl desaturase 1), an enzyme required for the lipid modification of the Wnts. Downregulation of miR-600 has been shown to promote the self-renewal of breast cancer stem cells whereas overexpression promotes their differentiation (El Helou et al., 2017). However, contrasting reports of promoter hypermethylation in case of multiple Wnt genes, including *WNT5A*, *WNT9A*, and *WNT10B*, suggest a potential tumor suppressive role (Yoshikawa et al., 2007; Ying et al., 2008; Galamb et al., 2016). The role of Wnt ligands in colorectal cancer has been reviewed in Nie et al. (2020). Promoter hypermethylation is also involved in silencing the Wnt negative-modulators, the DKK family, leading to hyperactivation of Wnt signaling in cancers (Nojima et al., 2007; Yu et al., 2009; Moskalev et al., 2012; Flanagan et al., 2017). Interestingly, *DKK1* promoter methylation has been reported in the later stages of cancer (Felipe de Sousa et al., 2011). Therefore, it might be assumed that it plays an important role in cancer progression instead of initiation. Promoter hypermethylation of *DKK3* has been reported in both colorectal cancer and breast cancer. However, its effect is not via suppression of LRP5/6 activity. Rather, it promotes membrane localization of β-catenin from the nucleus (Xiang et al., 2013). Promoter hypermethylation of *DKK2* has been reported but no correlation was observed with its expression (Silva et al., 2014). Apart from promoter methylation, *DKK1* promoter is also targeted by removal of activatory histone marks, such as H3Ac and H3K4me3 due to loss of p300 and recruitment of HDACs to its promoter, leading to reduced expression (Kim H.-Y. et al., 2015; Bao et al., 2017; Huang et al., 2013). DKK1 expression is also inhibited due to the upregulation of certain miRNAs, such as miR-375 and miR-376 (Taube et al., 2013; Cui et al., 2016). Silencing the Wnt antagonistic protein sFRPs by promoter hypermethylation resulting in the aberrant activation of Wnt signaling has been identified in diverse cancers with increased tumorigenic risk (Yu et al., 2019). Promoter hypermethylation of S*FRP1, 2, 4*, and *5* has been reported which correlates with the expression and cancer stage in case of breast cancer and colorectal cancer (Suzuki et al., 2002; Fujikane et al., 2010; Li et al., 2018). However, S*FRP3* does not harbor a CpG island in the promoter region (Suzuki et al., 2002). sFRP1 is also downregulated via multiple miRNAs in breast cancer (Yang et al., 2015). Downregulation of sFRP1 by hypermethylation has been shown to be essential for self-renewal of colorectal cancer stem cells (Li et al., 2018). S*FRP2* promoter hypermethylation occurs in HBV-associated hepatocellular carcinoma and breast cancer (Veeck et al., 2008; Yu et al., 2019; Xiang et al., 2021). Downregulation of WIF1 occurs by promoter hypermethylation in colorectal cancer, breast cancer, ovarian cancer and several other cancer types (Ai et al., 2006; Paluszczak et al., 2015; Hu et al., 2018). miR-603 is known to target WIF1 in glioma (Guo et al., 2014).

#### Receptors

Almost all members of the Wnt receptor family, such as FZD4, 6, 7, 8, and 10 have been reported to be regulated by different miRNAs in multiple cancer types including colorectal cancer, prostate cancer, breast cancer (Gong et al., 2014; Kim B.-K. et al., 2015; Jiang et al., 2016; Song et al., 2017; Li Z.-T. et al., 2020). In a few cases, upregulation of miRNAs regulating the negative regulators of FZD proteins can also contribute toward the severity of cancer. For example, miR-106b is upregulated in breast cancer which suppresses BRMS1L. BRMS1L is responsible for regulating the expression of FZD10. It recruits HDAC1 to *FZD10* which leads to promoter H3K9Ac (Gong et al., 2014). miR-130-30p, which is downregulated in triple negative breast cancer, acts as a Wnt antagonist by targeting multiple Wnt components, including FZD6 and LRP6 (Poodineh et al., 2020). Another lncRNA, GATA4-AS1 inhibits Wnt signaling in gastric cancer by reducing FZD4 expression. It recruits EZH2 and increases H3K27me3 occupancy at the promoter region (Ma et al., 2017; Li Z.-T. et al., 2020). LRP6 is also under the regulation of multiple miRNAs which are downregulated in colorectal cancer and breast cancer (Kim et al., 2011; Hata et al., 2017; Poodineh et al., 2020). MEST (mesoderm specific transcript) is a known regulator of LRP6 (Ruan et al., 2020). It blocks LRP6 maturation via glycosylation. Inhibition of MEST, therefore, enhances Wnt signaling (Ruan et al., 2020). MEST itself is targeted by miR-181 in ovarian cancer cells thereby promoting Wnt signaling. However, in another study, MEST was shown to be a positive regulator of the Wnt pathway. It was found to be under the regulation of zinc finger protein ZFP57, a transcription factor involved in maintaining DNA methylation in ESCs via its interaction with DNMTs. Promoter hypermethylation at *MEST* locus by ZFP57 was shown to be lost in breast cancer which led to enhanced Wnt signaling (Chen L. et al., 2019). Upregulation of the upstream positive regulators for LGR5 have been reported to serve toward cancer progression. For instance, loss of miR-363 promotes GATA6 expression and loss of miR-34 promotes ZNF281 expression, both of which are required for the expression of LGR5 in colorectal cancer (Hahn et al., 2013; Tsuji et al., 2014). However, promoter hypermethylation in case of LGR5 suggests a tumor suppressive role instead. Interestingly, the degree of methylation correlated with the grade of tumor (Schuebel et al., 2005; Felipe de Sousa et al., 2011; Su et al., 2015).

#### Destruction Complex Components

Adenomatous polyposis coli is the most frequently mutated gene in Wnt driven cancer cases, especially in the case of familial adenomatous polyposis. APC is also downregulated due to promoter hypermethylation, which can occur with or without mutations in the gene itself (Su et al., 1992, 1993; Arnold et al., 2004; Segditsas et al., 2008; Liang et al., 2017). It is also regulated by multiple miR-RNAs, such as miR-135 in colorectal cancer and miR-142 in breast cancer (Nagel et al., 2008; Isobe et al., 2014). miR-135b suppresses APC and is itself part of a positive feedback loop with TAZ (Shen S. et al., 2017). AXIN2 is often induced after Wnt activation and is a part of a negative feedback loop. It limits the duration of Wnt signaling. It is primarily found to be expressed in colon cells (Lustig et al., 2002). AXIN2 repression helps in prolonging the duration of Wnt signaling by blocking the negative feedback loop as well as increasing the duration of Wnt target genes expression (Lustig et al., 2002). Therefore, *AXIN2* is frequently targeted in colorectal cancer by promoter hypermethylation (Koinuma et al., 2006). Interestingly, AXIN2 is also downregulated by miR-34 in colorectal cancer which should inhibit Wnt signaling but miR-34 targets a number of Wnt activators as well, thus limiting the action of AXIN2 (Kim et al., 2013). Recently, miR-103/107 were also shown to promote colorectal cancer stemness by targeting AXIN2. miR-103/107 also promotes colorectal cancer by targeting LATS2. AXIN2 overexpression also led to nuclear accumulation of GSK3-β which would ultimately promote Wnt signaling (Kim et al., 2013; Chen H.-Y. et al., 2019). As expected, GSK3-β, βTrCP and CK1α, are downregulated in Wnt driven cancers, thereby promoting β-catenin stabilization. Table 2 summarizes the miRNAs reported to be responsible for negative regulation of these proteins in multiple cancers (Zhang et al., 2012; Li T. et al., 2016; Tan et al., 2016; Wang S. et al., 2016; Jin et al., 2017). DVL1, 2, and 3 are under the positive regulation by SIRT1 (a member of histone deacetylase family) in case of breast cancer (Holloway et al., 2010; Simmons et al., 2014). DACT (Disheveled Binding Antagonist of β-catenin) family of proteins are known negative regulators of Wnt signaling. DACT3 undergoes transcriptional repression due to the deposition of bivalent histone modifications H3K27me3 (repressive) and H3K4me3 (activatory) (Jiang et al., 2008).

Due to the dual role of YAP/TAZ in regulating the Wnt signaling pathway, there have been contrasting reports showing both tumor suppressive as well as oncogenic roles of YAP and TAZ. For example, miR-506, which targets YAP, is downregulated in colorectal cancer but upregulated in breast cancer (Hua et al., 2015; Krawczyk et al., 2017). The *YAP* promoter also shows aberrant hypermethylation in breast cancer (Real et al., 2018). However, the majority of studies point toward an oncogenic role of YAP and TAZ. Some of these have been listed in Table 2 (Shen et al., 2018; Yang M. et al., 2019; Yang et al., 2021). Hippo kinases and their upstream regulators are not a part of the Wnt signaling but it is important to mention them since these regulate the nuclear levels of YAP/TAZ and as a result, β-catenin. LATS has been reported as a tumor suppressor in colorectal cancer, head and neck squamous cell carcinoma, breast cancer and renal cell carcinoma. The promoter of *LATS1/2* undergoes hypermethylation (Okami et al., 2005; Steinmann et al., 2009; Chen et al., 2013; Chen K.-H. et al., 2014; Wierzbicki et al., 2013). Several miRNAs have also been reported to control the levels of LATS1/2 (Lee et al., 2009). Promoter hypermethylation has also been reported for *MST1/2* (Steinmann et al., 2009; Maille et al., 2019). MORC2 (microrchidia) acts as a negative regulator of Hippo signaling. MORC proteins are epigenetic readers as well as chromatin remodelers which help in DNA methylation (Pastor et al., 2014; Li S. et al., 2016). It forms a complex with DNMT3A and causes hypermethylation at the promoter regions of *NF2* and *KIBRA* which are upstream regulators of the Hippo pathway. Depletion of NF2 and KIBRA in turn increases the nuclear levels of YAP/TAZ and promotes stemness and oncogenicity in hepatocellular carcinoma (Wang T. et al., 2018).

#### Transcriptional Regulators

Promoter hypermethylation at *CTNNB1* gene is not observed frequently in colorectal cancer (Galamb et al., 2016). miR-34, which itself is a target of p53, regulates β-catenin. Loss of p53 in colorectal cancer and breast cancer in turn leads to upregulation of β-catenin (Kim et al., 2011). Some miRNAs responsible for regulating β-catenin levels are mentioned in Table 2 (Tian et al., 2014; Abedi et al., 2015). Interestingly, miR-520f-3p attenuates nuclear localization of β-catenin by targeting degradation of SOX9 mRNA in gastric cancer (Chen et al., 2020). JMJD1A, a histone demethylase, is responsible for upregulation of β-catenin expression in colorectal cancer. JMJD1A can also directly interact with β-catenin leading to its transactivation and upregulation of expression of multiple Wnt target genes, such as *MYC*, *CCND1* and *MMP9* (Peng et al., 2018). *TCF7L1* and *TCF7L2* promoters have hypomethylation in colorectal cancer (Guo et al., 2015). Several miRNAs regulating various members of the TCF/LEF family are downregulated in cancers (Table 2; Kim et al., 2011; Subramanian et al., 2014; Wang O. et al., 2018). Interestingly, the LEF1 gene has two promoters, P1 and P2. P1 is under the regulation of Wnt signaling, therefore upregulation of Wnt leads to increased expression of LEF1. Transcription from P2, on the other hand, produces a dominant negative form of LEF1. Transcription from P2 promoter is lost in case of breast cancer due to the repressive mark H3K9me3 mediated by YY1 (Yokoyama et al., 2010). Chromatin modification-induced alterations in Groucho expression are not frequent. However, downregulation of Groucho has been reported in colorectal cancer via the action of HDAC3 (Godman et al., 2008).

### Epigenetic Regulation Downstream of Wnt Signaling

Recent advances in cancer biology have identified the role of epigenetic regulation in cancers and numerous studies have identified mutations in the genes encoding both, the components and the regulators of Wnt signaling to aberrantly activate the Wnt signaling pathway in various cancers. In this section, we will elaborate upon the epigenetic modulations introduced by the Wnt pathway that promote cancer initiation as well as progression.

#### DNA Methylation

Multiple pathways and molecular players influence the outcome of the Wnt pathway. A study by Song et al. (2015) has shown that β-catenin interacts with DNMT1 and their interaction is required for mutual stabilization. Further, β-catenin complex with DNMT1 is a key requirement for DNMT1 dependent promoter methylation and Wnt/β-catenin signaling dependent target gene expression (Song et al., 2015). In another study, tumor suppressor- Na^+^/H^+^ exchanger regulatory factor 1 (NHERF1/EBP50), an adaptor molecule known to suppress Wnt signaling has been observed to be downregulated in colon cancer cells and is associated with decreased survival and increased intestinal tumor burden (Georgescu et al., 2016). Promoter methylation by DNMT1 has been implicated in reducing the expression of NHERF1/EBP50. Furthermore, Wnt signaling dependent upregulation of DNMT1 has been shown to be required to trigger hypermethylation of *NHERF1* promoter in colon cancer (Guo et al., 2018). Mechanistically, whether β-catenin recruits DNMT1 differentially on tumor suppressor genes and oncogenes, and what other molecular partners may be required to decide DNA methylation dependent and independent roles has not been fully explored. A recent study adds another layer of complexity by the Wnt signaling via crosstalk with various chromatin modifiers. The study shows that EZH2 dependent protein stability of LSD1, HDAC1, DNMT1, β-catenin, or SMAD2/4, via recruitment of deubiquitinase USP7, is key in suppressing neuronal genes and sustaining the expression of Wnt and TGFβ target genes in cancer cells (Lei et al., 2019). Understanding the role of β-catenin in DNMT1 regulation and recruitment would be crucial in developing future therapeutics.

#### Histone Modifications

The Wnt pathway employs diverse factors to regulate target gene expression and converges with epigenetic signaling at Wnt response elements through recruitment of epigenetic modulators. Upon Wnt stimulation, β-catenin translocates to the nucleus and interacts with the TCF family proteins by replacing co-repressors with subsequent recruitment of CBP/p300 HATs, thereby promoting H3 and H4 lysine acetylation. This reverses the HDAC-dependent chromatin compaction and induces molecular events to promote Wnt target gene expression (Parker et al., 2008). Although this study has emphasized on the role of β-catenin as an transcriptional activator, dependent on the recruitment of CBP/p300 HATs, it has been demonstrated that CBP/p300 can act as a bimodal regulator, regulating TCF/β-catenin interaction and also facilitating transactivation of β-catenin (Li et al., 2007). The ability of β-catenin to recruit multiple factors on promoters and the regulatory role of Wnt/β-catenin signaling in determining heterogeneity at the cellular level suggest that β-catenin could facilitate a poised chromatin state during developmental programs. Furthermore, information on how β-catenin regulated gene loci are selected as poised chromatin loci during the unfolding of developmental programs is still incomplete. Blythe et al. (2010) found that in *Xenopus*, prior to the midblastula transition during embryogenesis, Wnt/β-catenin signaling uncouples the activation of dorsal specific genes by establishing a poised chromatin state through recruitment of arginine methyltransferase PRMT2 at target gene loci. The PRMT2 catalyzes histone H3 arginine 8 (H3R8me2) asymmetric methylation. The advantage of such a poised nature of chromatin is to enable synchronous and rapid activation of tissue specific genes (Blythe et al., 2010). Another study showed that the C-terminal transactivation of β-catenin domain interacts with TRIPP/TIP60 and mixed-lineage leukemia (MLLI/MLL2) SET1-chromatin modifying complexes and recruits H3K4me3 at the promoters of target genes (Sierra et al., 2006; Zhu et al., 2019). These studies provide a mechanistic clue that during embryogenesis, pattern and combinations of these modifications could serve as critical molecular cues deciding tissue specific gene expression. Also, PRMT2-dependent poised state during early developmental stages could subsequently be followed by MLL1/MML2 driven gene activation in later stages. A recent study has shown that the dual pattern of histone modifications dependent on Spindlin1, a multivalent epigenetic reader, which potentiates Wnt/β-catenin signaling by recognizing the dual histone modification pattern ‘H3K4me3-H3R8me2’ (Su et al., 2014). Recently, Spindlin1 has been shown to drive the growth of colorectal carcinoma which have high levels of β-catenin. Spindlin1 is expressed in Lgr5^+^ intestinal stem cells and is required for self-renewal. It also promotes cancer stemness in a β-catenin-dependent manner (Su et al., 2014; Grinat et al., 2020). Previously, a report had shown that the MLL1/CBP/β-catenin complex promotes tumor initiation and metastasis in the head and neck squamous cell carcinoma by increasing the activation associated mark H3K4me3 at the promoters of target genes (Qiang et al., 2016). Interestingly, SETD7, a histone lysine methyltransferase, has been shown to be the part of the destruction complex itself. SETD7 mediated methylation of YAP aids in Wnt induced nuclear localization of β-catenin and thus is essential for intestinal regeneration and also plays a role in promoting tumorigenesis (Oudhoff et al., 2016). The studies demonstrating crosstalk of β-catenin transactivation with the epigenetic code provides new avenues to be tested in future for therapeutic intervention.

##### Wnt signaling and burst of chromatin reorganization

The genetic information on DNA is organized into the structural and regulatory framework of chromatin thereby allowing different machineries and signaling networks to switch off or switch on the gene expression. The major component of eukaryotic chromatin is the nucleosome comprising of 146 bp DNA wrapped around an octamer of core histones H2A, H2B, H3, and H4. The different machineries of transcription gain access to this complex structural framework by posttranslational modification of histones (Shilatifard, 2006; Berger, 2007; Fu et al., 2016). Depending upon the type of modifications and recruitment of modifiers, mediators and suppressors, gene activation or silencing is promoted (Kouzarides, 2007).

**FIGURE 4:**
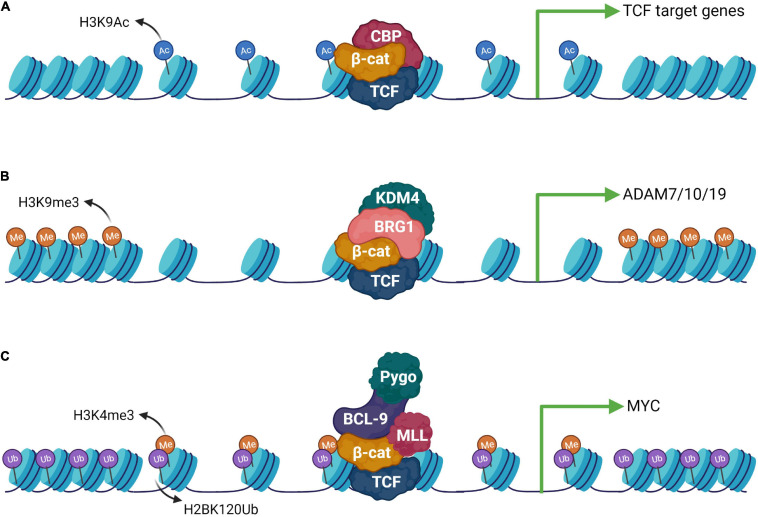
Epigenetic machinery downstream to the Wnt signaling pathway. **(A)** β-catenin can form complexes with different epigenetic modulators and activate gene expression upon Wnt activation. CBP is a well-known interaction partner of β-catenin which helps in opening up of the chromatin by catalyzing H3K9 acetylation. **(B)** Histone demethylase KDM4 is recruited via BRG1, an interactor of β-catenin which stimulates the expression of the ADAM group of genes. **(C)** β-catenin can also form an elaborate complex with the chromatin remodelers composed of the MLL complex, Pygo and Bcl-9 leading to H3K4me3 modification. This activity requires prior ubiquitination of H2BK120.

Wnt signaling drives molecular changes through the nuclear function of a prominent member of the ARM repeats containing protein, β-catenin. The central ARM repeats bound to TCF are critical for its function and their deletion completely abrogates β-catenin function (Xing et al., 2008). In the absence of nuclear β-catenin, TCF is bound to the co-repressor complex to stimulate compression of chromatin and suppress Wnt target gene expression (Cavallo et al., 1998; Brantjes et al., 2001; Courey and Jia, 2001). The N-terminal and C-terminal regions of β-catenin are transactivation domains known to interact with multiple cofactors to regulate Wnt target gene expression. The C-terminal domain (CTD) of β-catenin interacts with histone acetyltransferases (CBP or p300) resulting in chromatin modifications promoting the activation of Wnt target genes (Hecht, 2000; Takemaru and Moon, 2000; Courey and Jia, 2001). Upon Wnt stimulation, in CBP-dependent manner, nucleosomes are acetylated up to 30 kilobases with enhanced rate and saturated within 5.5 h, inducing a widespread wave of chromatin acetylation and re-organization (Figure 4A; Parker et al., 2008). The widespread use of acetylation and rapid activation of target genes is suggested to be dependent on the nature of TCF binding. TCF belongs to the high mobility group of proteins which are known to induce strong DNA bending upon binding. TCF induced bending facilitates the architectural framework for large chromatin regions to be organized together to stimulate a burst of chromatin reorganization dependent on β-catenin bound HAT (CBP) activity (Heintzman et al., 2007; Parker et al., 2008). This architectural framework of chromatin by the TCF/β-catenin/HAT complex provides a robust mechanism for rapid activation of Wnt target genes. The β-catenin CTD acts as a scaffold to recruit multiple diverse factors, such as, the SWI/SNF factors BRG1 and ISWI, HMTs, the Mediator component MED12, and the polymerase-associated factor 1 PAF1 (Waltzer et al., 2001; Kim et al., 2006; Mosimann et al., 2006; Sierra et al., 2006). These factors rearrange histone positions, modify the histones post-translationally and orient RNA Pol II, thereby providing the sequential exchange platform for remodeling chromatin architecture to induce rapid gene expression (Figure 4C; Mosimann et al., 2009). β-catenin has been shown to pre-pattern chromatin signatures during differentiation in an OCT4-dependent manner (Ying et al., 2015). Wnt signaling driven β-catenin acts as a molecular switch for the chromatin-associated high mobility group protein (HMG-17) to de-repress the inhibitory complex HMG-17/PITX2. The inhibitory complex HMG-17/PITX2 binds to specific chromatin regions primed for transcriptional activation and β-catenin forms a ternary complex with HMG-17/PITX2, thereby switching the inhibitory complex, modulating chromatin structure and inducing spatiotemporal expression of genes during embryogenesis (Amen et al., 2008; Ying et al., 2015). The histone methyltransferase and demethylase work in concert to stringently regulate gene expression. Protein lysine demethylases (KDMs) modify chromatin by demethylation of histones, especially repressive mark histone H3 lysine 9 trimethylation (Shi, 2007). The transactivation potential of β-catenin is very crucial for its nuclear function to target gene expression. Several studies have shown that β-catenin interacts with KDMs to epigenetically modulate target gene expression. For instance, β-catenin has been shown to interact with KDM3 on Wnt target genes inducing histone H3 lysine 9 (H3K9me2) demethylations. The β-catenin/KDM3 complex further drives MLL1-dependent H3K4 methylation to promote recruitment of BCL9 and Pygo2 to chromatin, thus promoting Wnt/β-catenin-dependent target gene expression (Li et al., 2019). In another study, KDM4 has been shown to physically interact with β-catenin and demethylated H3K9me3 on the promoters of Wnt target genes to promote target gene expression (Figure 4B; Peng et al., 2019). The complex nature of the cellular context requires multiple factors to work in concert for precise activation of target genes. For example, β-catenin recruits BRG1-dependent KDM4 isoforms on the promoters of Disintegrin and Metalloproteinase (ADAM) to regulate their expression in colorectal cancer cells (Sun et al., 2020). Thus, these findings establish that β-catenin recruits KDM to remove repressor marks and facilitate concomitant recruitment of activator complexes. However, the context-dependent and temporal nature of these β-catenin driven modulations require further investigation. The Wnt signaling-dependent activation of β-catenin and nuclear interaction with several transcription factors and chromatin acts as a molecular switch to regulate global gene activation. For example, β-catenin interacts with the chromatin organizer special AT-rich binding protein 1 (SATB1) and mediates the expression of specific set of genes during T helper-cell differentiation (Notani et al., 2010) and colorectal cancer progression (Mir et al., 2016), presumably via alterations in chromatin domain organization (Galande et al., 2007). The precise spatiotemporal activation of gene expression during development is a critical event dependent on chromatin organization and Wnt signaling directs the chromatin modifying machinery to establish a poised state at promoters (Shahbazian and Grunstein, 2007; Blythe et al., 2010). Wnt signaling has been shown to regulate differential developmental outcomes; however, it is not clear which mechanism governs this. Recently, Esmaeili et al. (2020) established that loss of chromatin architecture at Wnt target gene promoters is responsible for differential outcome of β-catenin bound chromatin without changing the inductive signal. The studies so far have established that Wnt signaling driven β-catenin modulates multiple aspects of gene regulation by acting as a signaling switch to regulate diverse cellular processes during development and tissue homeostasis.

#### Non-coding RNAs

Wnt signaling and microRNAs play essential roles in embryonic development and tissue homeostasis. MicroRNAs are involved in tumorigenic pathways. They play differential roles either regulating oncogenes or tumor suppressors to promote cancer progression (Xu and Mo, 2012). A recent study by Roman et al., has shown in cell-based experiments that microRNAs can modulate Wnt signaling by either regulating positive regulators or negative regulators of the Wnt pathway (Anton et al., 2011). Aberrant activation of Wnt signaling in various cancers regulate a wide range of gene expression at transcript level including microRNAs (Table 3). Oncogenic miRs-miR-182, miR30e, miR122, and miR21 are directly regulated by Wnt signaling in tumorigenesis (Liao and Lönnerdal, 2010; Lan et al., 2012; Chiang et al., 2013). In another study, miR215 and miR137 are repressed and miR708, miR135b, and miR3 are upregulated in *APC* mutant tumors (Necela et al., 2011). In gastric cancers, the expression of miR4739, miR210, miR135-5P, and miR12334-3P are significantly increased and the expression of miR20a-3p, miR-23b-5p, miR335-3p, miR423-5p, and miR455-3p is significantly reduced upon knockdown of β-catenin (Wang et al., 2009; Dong et al., 2015). In hepatocellular carcinoma, Wnt/β-catenin signaling upregulates miR770, miR183, miR96, and miR182 (Leung et al., 2015; Wu et al., 2016) and downregulates miR375 (Ladeiro et al., 2008). In breast cancer, miR182 and miR125b are upregulated by Wnt signaling and miR Let-7 is downregulated (Cai et al., 2013; Chiang et al., 2013; Liu et al., 2013). Aberrant activation of Wnt/β-catenin and microRNAs also involve autoregulatory feedback loop in cancers, for example, miR218 expression is induced by Wnt signaling and in turn, it augments the Wnt signaling pathway by targeting Wnt signaling inhibitors (SOST, DKK2, and sFRP2) (Hassan et al., 2012). Similarly, miR146a expression is induced by Wnt signaling in CRC, and in turn, it hyperactivates Wnt signaling by reducing the expression of Numb (Hwang et al., 2014). In summary, crosstalk between Wnt/β-catenin and miRs is crucial for the development of cancers and further understanding of this intricate signaling paradigm will provide insights for future therapeutics.

**TABLE 3:** miRNAs and lncRNAs regulated by Wnt pathway in various cancer types.

miRNAs			
Cancer type	Upregulated	Downregulated	References
**CRC**	miR-708, miR-31, miR-135b, miR-21, miR-182, miR574-3p, miR-30e	miR-215, miR-137	Liao and Lönnerdal, 2010; Lan et al., 2012; Chiang et al., 2013; Necela et al., 2011
**GC**	miR-20a-3p, miR-23b-5p, miR-335-3p, miR-423-5p, miR-455-3p	miR-1234-3p, miR-135b-5p, miR-210, miR4739, miR-122a	Wang et al., 2009; Dong et al., 2015
**HCC**	miR-770, miR-183, miR-96, miR-182	miR-375	Ladeiro et al., 2008; Leung et al., 2015; Wu et al., 2016
**BC**	miR-182, miR-125b	Let-7	Cai et al., 2013; Chiang et al., 2013; Liu et al., 2013
**IncRNAs**			
**Cancer Type**	**IncRNAs**	**Outcome**	**References**
**CRC**	KIAA0125	Downregulated	Yang Y. et al., 2019
**CC**	CALML3-AS1	Upregulated	Liu et al., 2019
**BC**	LUCAT1	Upregulated	Zheng et al., 2019
**LC**	LINC00673-v4	Upregulated	Lin et al., 2020
**LC**	AK126698	Downregulated	Fu et al., 2016
**Pituitary adenoma**	CLRN1-AS1	Downregulated	Wang et al., 2019
**Glioma**	SNHG17, MIR22HG, HOXC13-AS, LSINCT5, H19, BLACAT1, SNHG5, LINC01503, AGAP2-AS1, OIP5-AS1, DANCR, SNHG7, NEAT1, AB073614, MIR155HG, CCND2-AS1, CCAT2 and MALAT1	Upregulated	Han et al., 2020
**Glioma**	CASC7, Linc00320, TUNAR, MEG3, CASC2 and PTCSC3	Downregulated	Han et al., 2020
**Colon cancer**	CCAT2	Upregulated	Ling et al., 2013
**LC**	LINC00673-v4	Upregulated	Guan et al., 2019
**Liver cancer**	TCF7	Upregulated	Wang et al., 2015

*CRC, colorectal cancer; CLL, chronic lymphocytic leukemia; BC, breast cancer; HCC, hepatocellular carcinoma; OC, ovarian cancer; CC, cervical cancer; GC, gastric cancer; HNSCC, head and neck squamous cell carcinoma; RCC, renal cell carcinoma; LC, lung cancer; PC, pancreatic cancer; OS, osteosarcoma.*

Numerous studies have shed light on the Wnt signaling-dependent role of lncRNAs in diverse cancers (Table 3) such as colorectal cancer (Yang Y. et al., 2019), cervical cancer (Liu et al., 2019; Yang Y. et al., 2019), breast cancer (Zheng et al., 2019), non-small cell lung cancer (Lin et al., 2020), and brain (Wang et al., 2019). Multiple mechanisms are operational via long non-coding RNAs to differentially regulate Wnt signaling. For instance, a gene desert located upstream of the *MYC* gene is known to express Wnt related lncRNAs such as CCAT1-L, CCAT1-S, CCAT2, and CASC11 (Shen P. et al., 2017). CCAT2 (Colon cancer associated transcript 2) is known to enhance Wnt/β-catenin signaling activity by binding to TCF and increasing its transcriptional activity subsequently activating the cascade of downstream gene expression crucial for cancer progression and invasion (Ling et al., 2013; Shen P. et al., 2017). lncRNA LINC00673-v4 enhances TCF/LEF activity and increases the binding of DEAD-box helicase 3 X-linked (DDX3) and casein kinase 1 (CK1), thereby potentiating Wnt signaling (Guan et al., 2019). Apart from acting as transcription factors, lncRNAs are also shown to interact with chromatin modifying enzymes to promote spatiotemporal expression of target genes (Marchese and Huarte, 2014; Guan et al., 2019). For instance, the lncRNA 34a regulates miR-34a by recruiting repressor epigenetic machinery DNMT3a/PHB2 and HDAC1 at the promoter of miR-34a, thereby inducing DNA methylation and histone deacetylation to repress its expression (Wang L. et al., 2016). miR-34a is a key component in the Wnt signaling pathway and it regulates TCF7 expression (Chen W.-Y. et al., 2015).

In summary, lncRNAs act as regulatory switches to modulate the signaling outcome by targeting various crucial components of the pathway. The lncRNA-Wnt signaling module employs several mechanisms to regulate the expression of Wnt target genes. LncRNAs provide signaling scaffolds and act as transcription factors, chromatin co-modifiers and mediators. The significant involvement of lncRNAs in Wnt signaling driven cancers makes them attractive targets for drug discovery and cancer therapy.

## Therapeutics

Epigenetic regulation plays a dynamic role in Wnt signaling driven cancers. Epigenetic alterations are reversible by using pharmacological inhibitors. Multiple approaches have been employed for epigenetic therapy in cancers. These include DNA methylating inhibitors, histone methyltransferase inhibitors and HDAC inhibitors. To reverse aberrant DNA methylation acquired in cancers, DNMT inhibitors azacitidine and decitabine were developed and approved for acute myeloid leukemia (AML) (Derissen et al., 2013). Aberrant methylation of histone tails is linked with gene silencing in cancers. Several methyltransferases such as EZH2, SETD2 and Dot1L have been implicated in cancers (Albert and Helin, 2010; Hamamoto and Nakamura, 2016). Currently, tazemetostat is the most advanced therapeutic drug against EZH2 whereas valemetostat, CPI-1205, and CPI-0209 are in clinical phase 2 trials (Gulati et al., 2018). Inhibition of EZH2 reverses the silencing of genes induced by EZH2 hyperactivation. Another tested mode of epigenetic therapy is the reversal of histone deacetylation promoted by HDACs. Four HDAC inhibitors- vorinostat, romidepsin, belinostat, and panobinostat have been approved by the FDA (Bates, 2020). Inhibition of HDACs causes concomitant increase in HAT activity resulting in global increase in acetylation, subsequently relaxing chromatin and inducing transcription of genes responsible for promoting cell death (Zhao et al., 2020). The mechanism of HDAC inhibitors inducing cell death is not clearly understood and needs to be addressed in future to have a deeper understanding of their mechanism of action for therapeutic use. The epigenetic therapies have been shown to reduce cell proliferation, induce cell death in hematological malignancies (Altucci and Minucci, 2009), but they have not been seen to be effective in solid tumors (Graham et al., 2009). The HDAC inhibitors are mostly non-isoform selective and with recent advances in drug discovery, isoform specific and dual capability HDAC inhibitors have been extensively developed for cancer treatment (Peng et al., 2020). Current knowledge and complexity of how HDAC inhibitors work in the cellular context and combination therapies targeting the epigenome could be useful for designing future therapeutic interventions.

**FIGURE 5:**
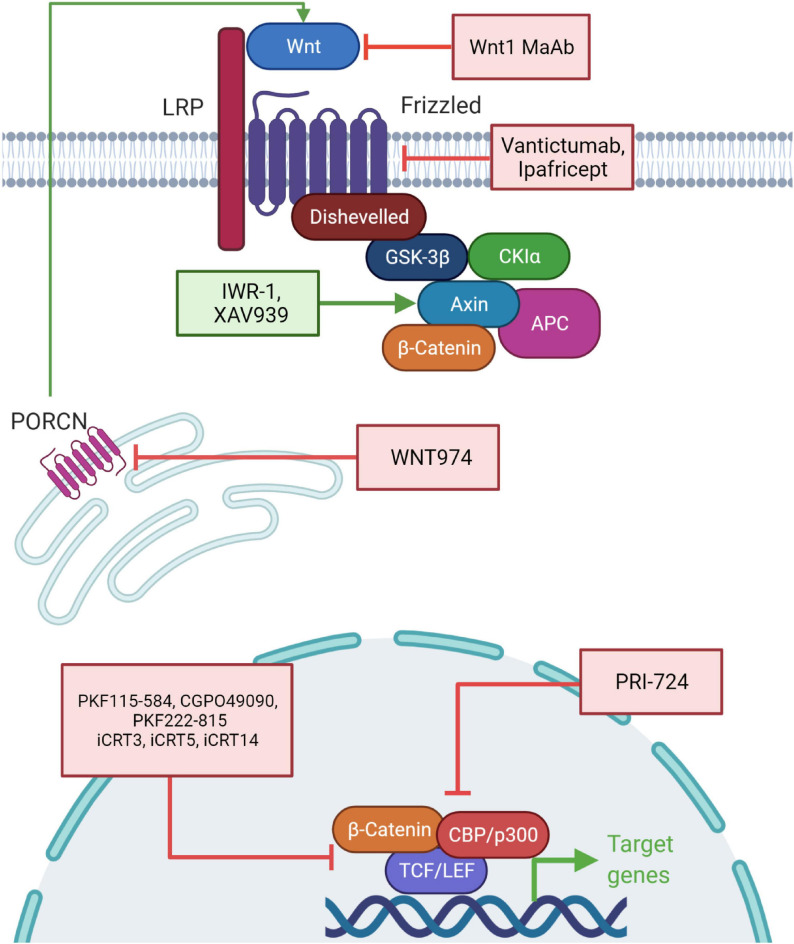
Chemotherapeutic interventions targeting the components of the Wnt signaling pathway. Drugs leading to the activation of the Wnt response inhibitors and repression of the Wnt response activators are being used as therapeutic agents for cancer therapy (or are under clinical trials). Wnt1 monoclonal antibody (MAb), Vantictumab and Ipafricept target the upstream regulators of the Wnt pathway. WNT974 blocks PORCN which prevents processing of Wnt ligands. IWR-1 and XAV939 act by activating AXIN, thereby stabilizing the Wnt destruction complex. PRI-724 acts by inhibiting the interaction of β-catenin with CBP/p300. PKF115-584, CGPO49090, PKF222-815, iCRT3, iCRT5, and iCRT14 act by inhibiting interaction of β-catenin with TCF/LEF.

Targeting the Wnt pathway is an attractive model for therapeutics and cancer therapy. Aberrant activation of Wnt signaling is associated with poor cancer outcomes and recent strategies involve targeting the pathway in diverse cancers (Figure 5). Inhibiting the stimulation of the Wnt pathway by using monoclonal antibodies against Wnt1 has been tested in many cancer cells and has resulted in reduction of downstream components and apoptosis (He et al., 2004; Esmaeili et al., 2020). Similarly, an anti-FZD antibody (Vantictumab) has been used to target the frizzled receptors and shown to reduce the tumor burden. This antibody was in clinical trials with patients of breast cancer and pancreatic cancer but eventually discontinued because of bone toxicity observed in these patients (Gurney et al., 2012; Smith et al., 2013). Ipafricept, a recombinant fusion protein consisting of FZD8 extracellular domain and human IgG1 FC fragment blocks Wnt ligands and xenograft assays have shown efficacy of this drug (Wall and Arend, 2020). It is in the first phase of clinical trials and is administered after chemotherapeutic agents (Fischer et al., 2017). Porcupine inhibitors, such as WNT974, inhibit Wnt secretion and are effective in reducing tumor growth. Porcupine catalyzes palmitoylation on Wnts required for their secretion. Currently, WNT974 is in phase 1 clinical trial (Koo et al., 2015; Agarwal et al., 2017). Another group of inhibitors such as IWR-1 and XAV939 target the stability of the β-catenin destruction complex, including inhibitors of tankyrase 1 and tankyrase 2; these induce AXIN protein stability. These inhibitors reduce Wnt signaling, EMT and angiogenesis (Arqués et al., 2016; Shetti et al., 2019).

The downstream signaling network of Wnt signaling pathway involves β-catenin nuclear signaling through interaction with various nuclear factors to drive target gene expression and tumorigenic phenotypic changes. Several attempts have been made to target this β-catenin and TCF interface to modulate target gene expression and induce regression of tumors. For instance, PKF115-584, CGPO49090, and PKF222-815 are fungal derived drugs and inhibit β-catenin’s interaction with TCF. These are currently in phase I and phase II of clinical trials (Lepourcelet et al., 2004). High throughput screening has identified three inhibitors, β-catenin responsive transcription inhibitor 3 (iCRT3), iCRT5, and iCRT14 specifically known to inhibit β-catenin and TCF interaction (Lepourcelet et al., 2004; Gonsalves et al., 2011). But clinical trials for these are not yet complete and potential side effects remain unknown.

The interaction of β-catenin with various co-factors is crucial for potentiating the Wnt pathway driven gene expression and associated cellular consequences, especially when the pathway is hyperactivated, has also been exploited to design drugs for cancer treatment. For instance, PRI-724 disrupts the interaction between β-catenin and its co-transcriptional activator CBP (Ko et al., 2016). Several natural compounds have also been used to treat Wnt signaling driven cancers such as resveratrol, curcumin, and Genistein (Cheng et al., 2019). The combinatorial use of epigenome modulating drugs along with conventional chemotherapeutic drugs will be required to treat epigenomic alteration driven cancers.

## Discussion

### Summary

In this review, we have provided an overview of the regulation of the Wnt signaling pathway in various cancer types, with a special emphasis on the crosstalk with the epigenetic machinery. Apart from undergoing alterations at the genetic level, the Wnt signaling pathway components are dysregulated due to various ncRNAs and chromatin modifiers. In turn, the Wnt effector β-catenin is also involved in various epigenetic processes due to its interaction with a multitude of epigenetic players. We have also provided an overview of the signaling pathways that work in conjunction or restrict the activity of the Wnt signaling pathway.

### Therapeutic Potential

Aberrant Wnt pathway activation seems to be the root cause for the development and progression of multiple cancers. However, even four decades after the discovery of the Wnt signaling, therapeutic intervention of this key pathway remains far from achieving its potential. Several factors seem to be responsible such as: the pathway is intricate and regulated by multiple combinations of Wnt ligands and their receptors, crosstalk of canonical and non-canonical pathways, and crosstalk with other signaling pathways. Further, multiple mutations of the Wnt signaling components cause constitutive activation of β-catenin, making it difficult to target the upstream players. Additionally, the physiological roles of the Wnt/β-catenin signaling are important for normal cellular functions. It is the combination of various signaling pathways that results in an imbalance in the rate of activation. Hence, future therapeutics will be aimed at optimizing the rate of activation and directing it toward maintaining the cellular homeostasis.

Using certain synthetic biochemical tags that could regulate levels of crucial components of the Wnt signaling pathway will be useful to develop future therapeutic intervention. Proteolysis-targeting chimeras (PROTACs) have emerged as an efficient technology to degrade proteins of interest *in vivo* (Sakamoto et al., 2001). One such strategy was employed recently using PROTAC chemistry to degrade β-catenin in Wnt signaling driven colorectal cancers. Membrane permeable PROTACs were designed to tether β-catenin with the ubiquitin system to induce controlled degradation of β-catenin to regress the tumor burden in *Apc* mutant mice (Liao et al., 2020). Similarly, PROTACs using biochemical inhibitors were designed to tag SGK3 kinase with VHL ubiquitin ligases to facilitate selective and efficient degradation of SGK3 in breast cancers compared to conventional inhibitors because of their sub-stoichiometric catalytic mode of action (Tovell et al., 2019). Recently characterized roles of the lncRNAs and chromatin in Wnt signaling regulation make them an attractive target for therapeutics in the future. Using a combination of PROTAC chemistry for crucial components of the Wnt pathway and chromatin, and nanoparticle based targeting of non-coding RNAs could enhance the chances of better therapies in the future. The precise and balanced stimulation of Wnt/β-catenin signaling governs cellular outcomes, tissue homeostasis and developmental paradigms and disruption of this balance may lead to a multitude of processes either in their favor or disfavor. Therefore, future therapeutics will depend on understanding this complexity employing multiple ways to curb the signaling imbalance.

### Future Perspectives: Open Questions

Wnt signaling crosstalks with several signaling and regulatory networks including epigenetic networks, mechano-sensing and environmental cues to modulate the expression of target genes crucial for developmental paradigms and tumorigenesis. Hence, holistic understanding of the molecular changes acquired during the initiation of Wnt signaling driven cancers is critical for disease etiology and future therapeutics. Identifying the pioneering molecular cues will be useful for developing better cancer therapies. The role of nuclear factors driving the Wnt/β-catenin signaling response raises hope of using drugs targeting these effectors. Obtaining a better picture of protein-protein interaction complexes regulating Wnt/β-catenin signaling will be helpful in identifying future therapeutic targets. Understanding the comprehensive picture of nuclear regulators and/or missing partner(s) crucial for convergence with other pathways will be essential for the treatment of Wnt signaling related diseases.

## Author Contributions

All the authors contributed to design the concept and structure of the review article, and approved the submitted version. AS and RM collected and analyzed the literature, and wrote bulk of the manuscript. AS prepared the figures. RM and SG provided critical inputs as the corresponding authors.

## Conflict of Interest

The authors declare that the research was conducted in the absence of any commercial or financial relationships that could be construed as a potential conflict of interest.

## Publisher’s Note

All claims expressed in this article are solely those of the authors and do not necessarily represent those of their affiliated organizations, or those of the publisher, the editors and the reviewers. Any product that may be evaluated in this article, or claim that may be made by its manufacturer, is not guaranteed or endorsed by the publisher.
